# A systematic comparison of community detection algorithms for measuring selective exposure in co-exposure networks

**DOI:** 10.1038/s41598-021-94724-1

**Published:** 2021-07-26

**Authors:** Subhayan Mukerjee

**Affiliations:** Department of Communications and New Media, Faculty of Arts and Social Sciences, National University of Singapore, Blk AS6, 11 Computing Drive, 117416 Singapore, Singapore

**Keywords:** Applied mathematics, Computational science, Statistics

## Abstract

The use of community detection techniques for understanding audience fragmentation and selective exposure to information has received substantial scholarly attention in recent years. However, there exists no systematic comparison, that seeks to identify which of the many community detection algorithms are the best suited for studying these dynamics. In this paper, I address this question by proposing a formal mathematical model for audience co-exposure networks by simulating audience behavior in an artificial media environment. I show how a variety of synthetic audience overlap networks can be generated by tuning specific parameters, that control various aspects of the media environment and individual behavior. I then use a variety of community detection algorithms to characterize the level of audience fragmentation in these synthetic networks and compare their performances for different combinations of the model parameters. I demonstrate how changing the manner in which co-exposure networks are constructed significantly improves the performances of some of these algorithms. Finally, I validate these findings using a novel empirical data-set of large-scale browsing behavior. The contributions of this research are two-fold: first, it shows that two specific algorithms, FastGreedy and Multilevel are the best suited for measuring selective exposure patterns in co-exposure networks. Second, it demonstrates the use of formal modeling for informing analytical choices for better capturing complex social phenomena.

## Introduction

A significant body of literature has emerged in recent years that uses network analytic techniques to understand how audiences navigate media environments ^1–7^. These techniques typically involve the construction of *co-exposure* ^2^ or *audience overlap*^4^ networks from behavioral trace data, which allows researchers to effectively model media consumption patterns in a wide variety of contexts. When used in conjunction with methods such as community detection, it enables certain theoretical notions of macro-level audience behavior such as selective exposure to information, polarization in news consumption patterns, and audience fragmentation to be effectively operationalized.

While such methods have seen widespread application in the social sciences for understanding the behavior of media audiences, their implementation rarely follows a strategy that is grounded in network theory or in principles that are agreed upon a priori. Unlike the construction of these networks, which usually follows a fixed methodology (the conceptual projection of a bipartite network into an audience overlap network), the analytical choices for investigating the network are often left to the discretion of the researcher. A prominent example can be found in the application of community detection algorithms, which are widely used to identify clustering, and in turn, make inferences regarding the level of selective exposure and polarization in media consumption patterns. The lack of any systematic comparison of how different community detection algorithms perform on co-exposure networks however means that it is possible that the inferences drawn from such analyses are artefacts of the analytical choices the researcher makes. In one example ^1^, the authors report differences in the performances of the different algorithms in the Online Supplementary Information even as they base their main conclusion on one of them. In another example ^3^, the authors find high concordance between the performances of only two community detection algorithms, and lower concordance between others. However, it is only one of the algorithms that they use to ground the rest of their analyses on. Choices such as these are endemic in the literature, but they are neither motivated by a systematic comparison of the various methods, nor rooted in rigorous theoretical principles. Nonetheless, they are instrumental in furthering our knowledge about the phenomena they are used to investigate. This makes it difficult to draw more general inferences about the theoretical constructs that these methods purport to capture, precluding any possibility for comparison across the contexts in which they are applied.

While analysis of co-exposure networks is a relatively new phenomenon, research on media co-exposure has a long tradition with advertisers in the United States using metrics capturing co-exposure to television programs to understand their audiences since the 1960s ^8–10^. Similar techniques were later used to investigate news exposure patterns and its relationship with political attitudes and behavior of the audiences. With the rise of online audiences these techniques have gradually lent themselves to the behavioral trace data that digital platforms have made possible. This has resulted in a large number of studies that use online web-browsing data or social media data to construct co-exposure or audience overlap networks to understand online media co-consumption patterns.

A co-exposure (or audience overlap) network is defined as a network with nodes representing media outlets, and edges capturing the number of shared consumers between pairs of outlets. Conceptually, it is the *media outlet projection* of a bipartite network that has two kinds of nodes, media outlets and media consumers, with edges between them implying an interaction between the corresponding outlet and consumer. This interaction could be a website visit, a page view, a social media “reaction” or a share. While originally used in conjunction with web-browsing data ^4,6,11,12^, the use of such networks has grown to witness application with social media data as well. Examples of the former include studies that used data sources such as the Nielsen TV/Internet Convergence Panel to construct a network with binary edges depending on whether or not the overlap between two outlets was higher than random ^6,11^. More recent studies have constructed similar co-exposure networks at a national ^4,13^ as well as at a global scale ^7,14^ using other online panel data sources such as ComScore.

With social media data, similar approaches have been used to understand selective exposure to partisan information on social media platforms. One study, for instance, used the Facebook graph API to build co-exposure networks capturing the interaction of Facebook users with Facebook pages that posted news about Brexit ^3^. The researchers then used community detection algorithms to find two clusters of users, who interacted with Brexit news on Facebook in significantly different ways. A larger study of news consumption on Facebook ($N = 376$ million) used community detection on a similar co-exposure network of 920 media outlets to show that even outside of political news, audiences exhibit selectivity in news consumption patterns ^1^. A more recent study constructed coexposure networks of Twitter audiences to understand the prevalence of exposure to fake news in the build-up to the 2016 Presidential Election ^2^. The authors used community detection algorithms to conclude that fake news reached a very niche and specialized audience, and did not feature prominently in mainstream news consumption behavior, at least among American voters who were active on Twitter. Yet other studies have used co-exposure networks of Twitter audiences to understand media consumption in contexts outside the US, such as in the UK ^15^ and in Asia ^16^.

While community detection has been used as methodological tool in a variety of other contexts as well ^17^, it is its applications in the field of media consumption that has been proven to be particularly fecund ^1–3,15^. This is because the intuition behind community detection - that strongly connected or closely clustered media outlets belong to their own community, which is structurally distinguishable from the rest of the network - lends itself to the idea of selective exposure particularly well. It allows researchers to formally capture the phenomenon that most people tend to preferentially choose certain media outlets to others, which itself has a long tradition in media studies and communication research ^18–20^.

However, a large number of community detection algorithms exist in the network science literature ^21,22^, and many of them fundamentally differ in how they operate ^23^. As a result, they often reveal completely uncorrelated community structures when applied to the same given network. Therefore, for application to co-exposure networks, which of the many community detection algorithms to use, and how their performances compare to each other remains an open question. In fact, the choices made in existing studies are largely arbitrary and driven by convention, instead of being informed by any systematic comparison. While there are papers that have compared different community detection algorithms in networks *more generally*, none has, to the best of my knowledge, looked at how the different algorithms perform *specifically* on co-exposure networks. This paper seeks to answer that question by simulating the behavior of news seekers (“agents”) in an artificial media environment, and generating a range of theoretical co-exposure networks. Then, it seeks to appraise the effectiveness of the most widely used community detection algorithms by comparing the community structures they reveal with a “ground truth” or expected community structure that is determined before the simulation is run. As is expected, the performances of the various algorithms vary greatly, with many of the algorithms failing to reveal any community structure at all. However, some algorithms work demonstrably better than the others. This paper also proposes an alteration to the method used for constructing the co-exposure networks, and demonstrates how the typical method for constructing co-exposure networks discards valuable information that otherwise, significantly enhances the performances of some of these algorithms. Finally, it shows how the performances of these algorithms, as predicted in the theoretical networks, are favorably replicated on a large real world co-exposure network data set as well. The study therefore, uses network theoretic considerations and formal modeling of a social phenomenon to assess the effect that methodological choices have in capturing that phenomenon. Using both theoretical predictions, as well as empirical validation, it lays out analytical recommendations for future scholars seeking to analyze co-exposure networks.

## Methods

### Model specification

The universe in this model comprises a set of $n_1$ media outlets $\mathscr {M} = \{m_1, m_2,\,\ldots\,m_{n_{1}}\}$, and a set of $n_2$ agents (media consumers) $\mathscr {A} = \{a_1, a_2,\,\ldots\,a_{n_{2}}\}$ who have the option to visit any or none of the $n_1$ media outlets. Corresponding to the set of media outlets is a reputation vector $\mathscr {R} = \{r_1, r_2,\,\ldots\,r_{n_{1}}\}$ with $r_{i}$ capturing the reputation of the media outlet $m_i$ The elements in this vector are drawn from a power-law distribution with exponent $\alpha$. This serves two purposes: first, the elements of $\mathscr {R}$ capture the inequality in prior popularity and reputation of the various outlets. and two, $\mathscr {R}$ serves as a weight vector, when probabilistically assigning outlets to an agent to visit, allowing agents to be more likely to visit a few reputable outlets than a large number of less reputable ones. Further, there is set of $n_3$ *types* of media outlets and agents $\mathscr {T} = \{t_1, t_2,\,\ldots\,t_{n_{3}}\}$, and a population level parameter $\rho$ that determines the extent of randomness in the agents’ media consuming behavior. When $\rho$ is 0, agents behave in a fully selective manner: all the media outlets they visit are of the same type as themselves, indicating a hypothetical condition of complete selective exposure When $\rho$ is 1, agents behave in a completely random manner, and can visit any media outlet in the universe, irrespective of the type. For any value of $\rho \in (0,1)$, the agents visit the corresponding fraction of outlets randomly, and the rest selectively. For example, for $\rho = 0.6$, $60\%$ of the outlets the agents visit are random, $40\%$ are selective. Finally, because people’s media consumption habits aren’t normally distributed but skew positive, the number of outlets each agent *i* visits, $v_i$ is drawn from a right skewed normal distribution $N(\mu , \sigma , k)$ where the skewness *k* is yet another model parameter. This ensures, that in line with prior empirical findings, only a minority of the agents visit a large number of outlets, while majority visit few of them. The full model is therefore, completely specified by the parameter set $\{\mathscr {M}, \mathscr {A}, \mathscr {T}, \alpha , k\}$. The implementation of the model specification is depicted in the InitUniverse procedure in the pseudocode supplied in the SI.

### Simulation

The model is simulated by iterating through every agent $a_i \in \mathscr {A}$ and assigning to them $v_i \in N(\mu , \sigma , k)$ media outlets as the ones they visit. The assignment is done by using weighted sampling, where each outlet $m_i$ is chosen with probability $r_i$. This is to ensure that every agent has a greater chance of visiting media outlets with higher values of $r_i$, so that final audience size of the media outlets resemble a power-law distribution.

Every simulation of this model yields a bipartite graph $\mathscr {G}(\mathscr {M},\mathscr {A},\mathscr {E})$ over the disjoint sets of $\mathscr {M}$ and $\mathscr {A}$ where an edge $e \in \mathscr {E}$ between $m_i \in \mathscr {M}$ and $a_j \in \mathscr {A}$ exists if agent $a_j$ visited media outlet $m_i$. Projecting this graph onto the vertex set $\mathscr {M}$ gives the weighted co-exposure network $\mathscr {G'}(\mathscr {M}, \mathscr {E}')$. Each weighted edge $e' \in \mathscr {E}'$ in $\mathscr {G'}$ between two media outlets captures the number of agents who visited both outlets. Each such weighted co-exposure network serves as my unit of analysis, on which I apply the various community detection algorithms and measure selective exposure.

**Figure 1:**
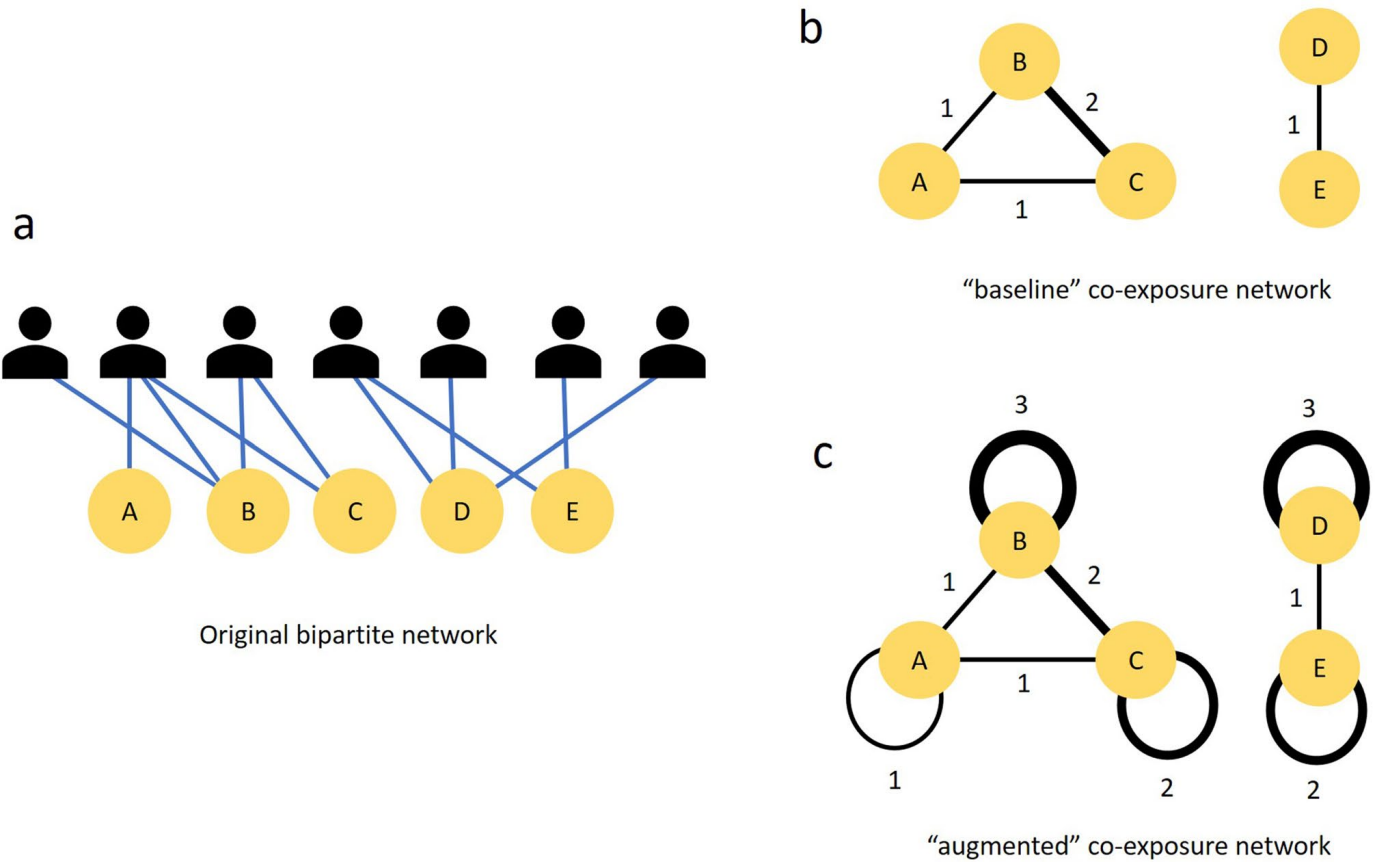
The construction of baseline and augmented co-exposure networks. Panel a shows the original bipartite network with each edge indicating that an agent visited an outlet. The outlet projection of this bipartite network is shown in Panel b (“baseline”). The edges here indicate co-exposure between pairs of outlets, with the weight of the edge capturing the shared audience between them. Panel c shows how the co-exposure network is augmented by adding self-loops with weights equal to the size of the audience of the respective node.

Formally, the adjacency matrix of this weighted network (one-mode projection) $\mathscr {G'}(\mathscr {M}, \mathscr {E}')$ is given by $\mathbf {B}^T\mathbf {B}$, where $\mathbf {B}^T$ is the transpose of the incidence matrix $\mathbf {B}$ of the original bipartite graph $\mathscr {G}(\mathscr {M},\mathscr {A},\mathscr {E})$. The usual definition for one-mode projections of bipartite networks ^24^ requires setting the main diagonal elements of $\mathbf {B}^T\mathbf {B}$ to 0. However, Arenas et al. ^25^ have shown that the community structure an algorithm reveals can be extremely sensitive to the values of this main diagonal of the adjacency matrix. These main diagonal values, in fact, represent weighted self-loops on the network vertices. Other studies have demonstrated that the addition of self-loops to the vertices of complex networks alters the community structure that a modularity-optimizing algorithm reveals by effectively changing the null model of the modularity of the network ^26–28^. Tweaking the weight of the self-loop and thereby altering the null model, changes the modularity and results in these algorithms performing differently and uncovering different community structures. With co-exposure networks, which are one-mode projections of bipartite networks, there is no need to tweak the weight of the self-loops, because the main diagonal values of $\mathbf {B}^T\mathbf {B}$ do indeed represent weighted self-loops, capturing the number of agents who visited each outlet. From a purely intuitive standpoint, it also makes sense to retain the elements of the main diagonal of the adjacency matrix, simply because it could be beneficial for the algorithm, as opposed to discarding them and potentially losing useful information. Therefore, I report two sets of results for each community detection algorithm I use. The first, with the main diagonal elements of $\mathbf {B}^T\mathbf {B}$ set to 0 (I call this the “baseline” network); and second, *without* setting the main diagonal elements of $\mathbf {B}^T\mathbf {B}$ to 0 (I call this, the “augmented” network), and compare the effect that retaining the self loop has on the performances of the algorithms. The logic of constructing the “baseline” as well as the “augmented” networks are visually depicted in Fig. 1. The pseudo-code implementations of the model simulation and network construction are provided in the SimulateAgentBehavior and ConstructNetwork procedures respectively, in the SI.

## Results

For each simulation, I apply eight different community detection algorithms on both, the baseline as well as the augmented network, thereby yielding sixteen resultant community substructures per simulation. The eight algorithms I choose are available in the “igraph” package, a popular network analysis library with implementations in R, Python, and C++. The eight algorithms along with their brief descriptions are as follows:

1. **Edge-betweenness** (EB) ^29^: this algorithm works by iteratively removing the edge with the highest edge betweenness centrality, thereby isolating network clusters that would otherwise be connected to each other.
2. **FastGreedy** (FG) ^30^: this algorithm operates by trying to find a partition of vertices by optimizing the modularity score of the network in a greedy manner.
3. **Infomap** (IM) ^31^: an information-theoretic technique that identifies network communities by simulating the flow of information through the network structure.
4. **Louvain or Multilevel** (ML) ^32^: an iterative algorithm that assigns vertices to communities in order to progressively increase the modularity score of the network.
5. **Leading Eigenvector** (LE) ^33^: this algorithm operates in two steps. First, it calculates the eigenvector of the modularity matrix for the largest positive eigenvalue. Then, it separates the vertices into communities based on the sign of the corresponding element in the eigenvector.
6. **Label Propagation** (LP) ^34^: an iterative algorithm that first randomly assigns labels to every vertex, and then keeps reassigning labels to every vertex based on the labels of its nearest neighbors, till reaching convergence.
7. **Spin-Glass** (SG) ^35^: this algorithm borrows ideas from statistical mechanics and identifies communities using a spin-glass model.
8. **WalkTrap** (WT) ^36^: a widely used algorithm that simulates the path of a random walker on the network over a long period of time, under the intuition that the walker will tend to get trapped within dense communities.

“igraph” gives a ninth function to implement an “optimal” modularity optimization algorithm but this algorithm did not converge for values of $\rho > 0.5$, and was therefore dropped.

To evaluate the performance of a community detection algorithm, the revealed community structure is usually compared with a “ground truth” that is known *a priori*. In most cases, this ground truth is informed by the use the LFR benchmark  ^37^, which allows one to generate artificial networks with pre-determined community structures, that are comparable to the networks one is analyzing. In this case however, the “ground truth” is woven into the model specification itself. We know *a priori* that there are $n_3$ types of media outlets. Each media outlet should, for all values of $\rho < 1$, on average, share stronger overlap with outlets of the same type, than with outlets of a different type, giving us a clear idea of the theoretical community structure that the algorithms should ideally reveal. This community structure would in turn, reflect the underlying selective exposure patterns that the simulation would have created. I leverage this knowledge and assess the performance of the algorithms using this pre-determined classification of media outlets as the benchmark. To formalize the performance metric, I use the Normalized Mutual Information (NMI) score ^38^ to compare the similarity between the vector of labels produced by the algorithm ${C} = \{c_{m_{1}}, c_{m_{2}},\,\ldots\,c_{m_{n_{1}}}\}$ and the vector of outlet types, determined prior to the simulation ${T} = \{t_{m_{1}}, t_{m_{2}},\,\ldots\,t_{m_{n_{1}}}\}$. The NMI value is given by equation (1):

1 $$\begin{aligned} NMI(C,T)= \frac{-2 \sum _{i=1}^{C} \sum _{j=1}^{T} N_{i j} \log \left( N_{i j} N / N_{i o} N_{o j}\right) }{\sum _{i=1}^{C} N_{i \circ } \log \left( N_{i o} / N\right) +\sum _{j=1}^{T} N_{o j} \log \left( N_{o j} / N\right) } \end{aligned}$$

Here, *N* is the confusion matrix with rows corresponding to the outlet types assigned prior to the simulation, columns corresponding to the community labels produced by the algorithm, and $N_{ij}$, the number of outlets of type *i* assigned to community *j*. The row sums and columns sums are denoted by $N_{io}$ and $N_{oj}$ respectively ^23^. Note that the vectors *C* and *T* have the same cardinality (equal to the number of outlets $n_1$) as they represent the community assigned to, and the pre-determined type of each of the $n_1$ media outlets in $\mathscr {M}$ respectively. $NMI(C,T) = 1$ when the algorithm produces a community label vector *C* identical to *T*, and 0 when they are independent and orthogonal. As Vinh et al. ^39^ show, the normalization can be done in various different ways, and the R function *NMI* in the *aricode* package, allows one to choose the *NMI* variant. I report the default *NMI* values (corresponding to $variant = max$ in the R function) in the main text, and report the other variants in the SI.

For a given set of model parameters $\{\mathscr {M}, \mathscr {A}, \mathscr {T}, \alpha , k\}$, I run 100 simulations for different values of $\rho$ ranging from 0 to 1 in steps of 0.1. For each simulation, I compute eight pairs of *NMI* values corresponding to the eight algorithms, applied first to the baseline network, and then to the augmented network (i.e. with the self-loops).

### Comparison on artificial networks

**Figure 2:**
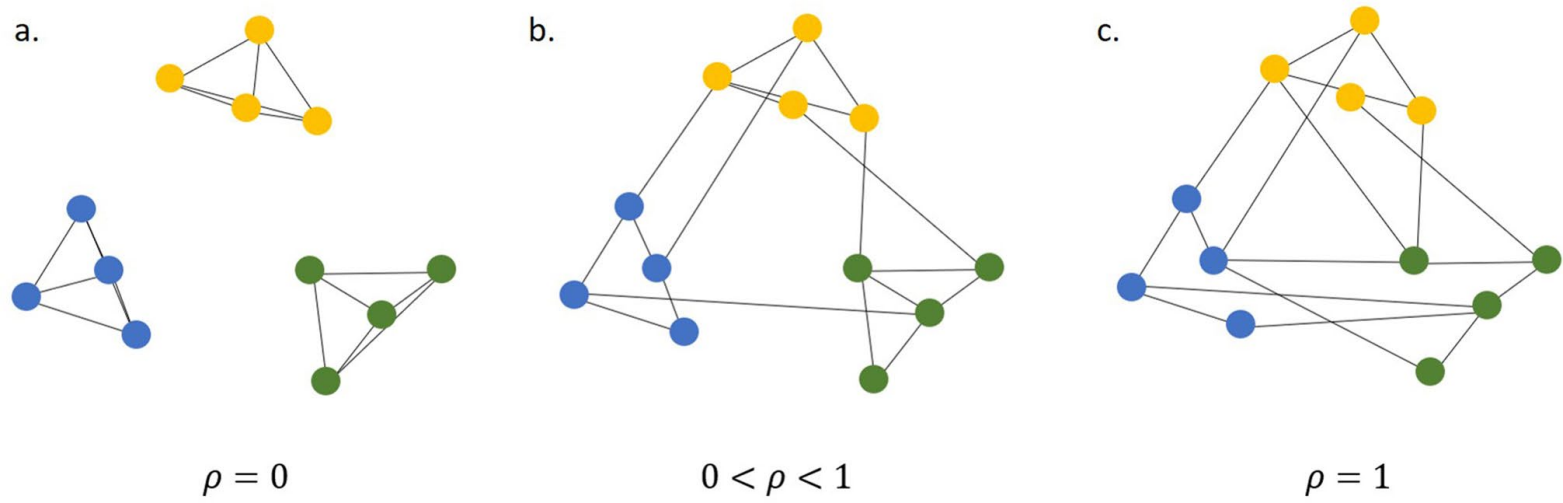
Illustration of the effect of $\rho$ on network structure using a toy synthetic network. Synthetic co-exposure networks at 3 values of $\rho$ are shown. The colors denote the types of media outlets. When $\rho = 0$, all agents visit outlets of the same type as themselves, creating zero co-exposure between different types of outlets (**Panel a**). When $0< \rho < 1$, all agents visit a fraction ($= \rho$) of the outlets they visit, randomly, and the remaining fraction ($= 1 - \rho$), selectively, i.e. of the same type as themselves. This creates some connections of co-exposure between outlets of different types (**Panel b**). As $\rho$ is increased, the number of connections between different types of media outlets increases, as the agents’ behavior becomes less selective, and more random. When $\rho = 1$, all agents visit all the outlets they visit, randomly, creating instances of co-exposure between outlets of different types as likely as instances of co-exposure between outlets of the same type (**Panel c**).

The community structure that should *theoretically* emerge upon running the simulation depends crucially on the value of $\rho$ that controls the extent of randomness in the behavior of the agents. For $\rho = 0$, because there is no randomness in the agents’ behavior, all agents only visit the same type of outlets as themselves. In other words, there is no co-exposure between outlets of different types as no agent visits outlets of more than one type. As $\rho$ is increased, the behavior of the agents become less and less selective till at $\rho = 1$, their behavior is completely random. Thus, theoretically, the community structure will be most prominent at $\rho = 0$, with every community (corresponding to every type) completely separated from the others. The community structure would gradually become less and less prominent as $\rho$ is increased as more and more co-exposure occurs between outlets of different types, till, eventually, no structure would emerge owing to the high degree of randomness in the agents’ behavior. The transformation in the structure of a toy network at three values of $\rho$ are shown in Fig. 2.

**Figure 3:**
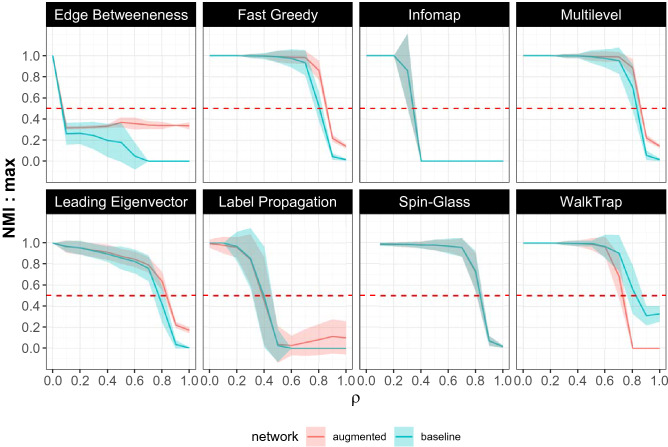
The performances of the 8 community detection algorithms are shown on the “baseline” (cyan) and “augmented” (pink) networks respectively. The y-axis tracks the Normalized Mutual Information value for each algorithm for every value of $\rho$, that determines the extent of randomness in the agents’ behavior. The solid line tracks the mean NMI score for the 100 simulations for every value of $\rho$, while the ribbons indicate the standard deviation around the mean. As $\rho$ increases, *NMI* values drop. Fast Greedy, Multilevel, WalkTrap and Spin-Glass outperform the others even when the randomness is substantial. With the augmented networks, while the performances of FG and ML are significantly enhanced, that of WT decreases.

Figure 3 shows the results of simulating the model with the following parameter values: $n_1 = 100$ outlets, $n_2 = 1000$ agents, $n_3 = 5$ types, $\alpha = 3$, and $k = 3$. For each value of $\rho$, the simulation was run 100 times. The mean NMI scores along with their standard deviations are visualized. The pink lines and ribbons correspond to the *NMI* values on the “baseline” networks, while the cyan lines and ribbons correspond to the “augmented” networks with self-loops. When $\rho = 0$, all the algorithms are able to to reveal the underlying community structure perfectly ($NMI = 1$). This is because, in the absence of any random behavior, the outlets of different types share no co-exposure with each other. In other words, the only edges that exist in the network are between outlets of the same type, producing a network with $n_3 = 5$ isolated and clearly identifiable communities. As $\rho$ is increased, the increased randomness in the agents’ behavior reduces the ability of the algorithms to reveal the perfect community structure and the *NMI* values gradually decline. At $\rho = 1$ almost all the *NMI* values drop to 0. The ones that still remain positive are owing to the algorithms overfitting the data, and often (for example, in the case of WalkTrap on the “baseline network”) assigning each node to its own community. This problem is fixed by the use of the *scaled* *NMI* ^40^ score that penalizes algorithms that produce a large number of communities. Scaled *NMI* is calculated as shown in Equation 2:

2 $$\begin{aligned} SNMI = e^{-\frac{|R-S|}{R}} \times NMI \end{aligned}$$

**Figure 4:**
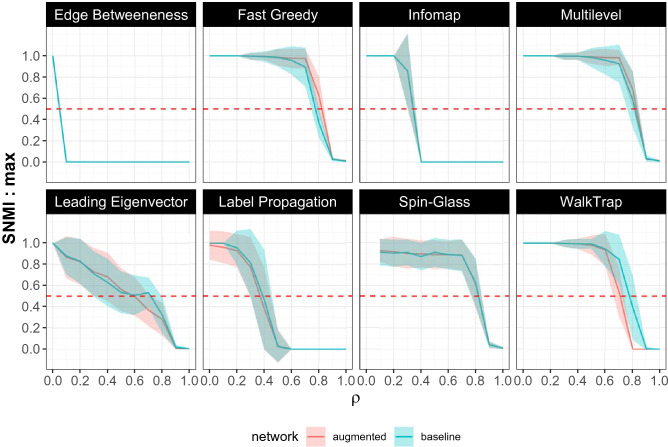
The performances of the 8 community detection algorithms are shown on the “baseline” (cyan) and “augmented” (pink) networks respectively. The y-axis tracks the *scaled* Normalized Mutual Information (SNMI) value for each algorithm for every value of $\rho$, that determines the extent of randomness in the agents’ behavior. The solid line tracks the mean SNMI score for the 100 simulations for every value of $\rho$, while the ribbons indicate the standard deviation around the mean. As $\rho$ increases, *SNMI* values drop. Fast Greedy, Multilevel, and Spin-Glass outperform the others even when the randomness is substantial. With the augmented networks, While the performances of FG and ML increase, that of WT decreases.

In the above equation *R* is the true number of communities, while *S* is the number of communities the algorithm predicts. When $S = R$, the numerator of the exponent becomes 0, and the scaling-factor becomes 1. When $S>> R$, the scaling factor (and consequently, the score) becomes vanishingly low. The results using the scaled *NMI* scores are shown in Fig. 4.

The algorithms that perform the best on the “baseline” networks are Fast Greedy (FG), Multi-Level (ML), and Spin-Glass (SG) while Edge-Betweenness (EB), Infomap (IM), and Label Propagation (LP) perform the poorest. This is evidenced by the drastic drop in *NMI* values for relatively low levels of randomness in the agents’ behavior $(0.1< \rho < 0.5)$ with EB, IM, and LP. FG, ML, SG on the other hand, are able to achieve relatively high values of *NMI* scores even when $0.5< \rho < 0.7$.

Their performances on the “augmented” network reveals a further nuance. ML and FG, which were already out-performing the other algorithms, report even higher *NMI* scores when the network is augmented with self-loops. Non-parametric Wilcoxon’s Rank Sum test between the *NMI* scores on the “augmented” networks and the *NMI* scores on the “baseline” networks demonstrate that the former are significantly higher $(p < 10^{-3})$ than the latter for $0.7< \rho < 0.9$. For $\rho < 0.7$, there is no significant difference because both ML and FG often yield perfect *NMI* scores of 1 on both the baseline and augmented networks. For SG, LP, IM, and Leading-Eigenvector (LE), “augmenting” the network with self-loops has no effect, while for WalkTrap (WT), the addition of self-loops significantly *lowers* the *NMI* scores.

The analyses demonstrate that at least on the artificially generated networks, not only do FG and ML out-perform the rest, but that their performances can be further enhanced by adding self-loops to the nodes of the network with weights equal to the size of the audiences of the nodes. Thus, contrary to usual the definition of one-mode projections ^24^, *not* setting the main diagonal elements of $\mathbf {B}^T\mathbf {B}$ to 0 while projecting the bi-partite network and constructing the co-exposure network, allows us to uncover a more accurate community structure. These results are robust to the values of skewness paramter *k*, being qualitatively identical for a wide range of values. The SI reports the results for $k = 1$ and $k = 15$. For $\alpha$, however, the performances of all the algorithms reduce substantially for values $< 2$. Their qualitative order remains very similar, even as they all under-perform. Results for $\alpha = 1$ and $\alpha = 2$ are reported in the SI. It should be noted here that very rarely do real world networks, whose degree distributions follow the power-law distribution, have exponents lower than 2 or greater than 3. In the next section, I show that these findings are favorably replicated in the case of a real-world empirical network as well.

### Comparison on a real-world network

To test whether the comparative analysis using the artificial networks mimic the findings when used with real world data, the primary challenge lies in getting a co-exposure network dataset with a clear community structure that is known a priori. For validation purposes, I therefore used a large scale dataset of desktop web-browsing behavior of a representative panel of Indian internet users, obtained from the media analytics firm, ComScore. The ComScore Indian panel provided the perfect context to appraise the performance of the algorithms because of the high degree of linguistic diversity in the country. A 2009 World Report by UNESCO, puts India at 9th in the world in terms of linguistic diversity with a Linguistic Diversity Index of 0.930. In contrast, the United States, France, Germany, and the UK rank 116th (LDI: 0.353) 128th (LDI: 0.272), 139th (LDI: 0.189), and 155th (LDI: 0.139) respectively ^41^. The high linguistic diversity results in the existence of large and dedicated news reading audiences that are defined along socio-cultural and ethno-linguistic divisions. In other words, India is a country that is characterized by a high-degree of selective exposure to media because of the large number of languages that exist in the country, and the large number of people who are fluent in their own regional vernacular. This makes substantial sections of Indian society selectively consume news (their own “regional” media) in their own language ^42,43^. English media, on the other hand, while more widespread *across* the country, doesn’t penetrate the rural swathes within every state, and remains the choice of only the upper-class elites across the nation.

I use the ComScore data to first construct a “baseline” co-exposure network with media outlets constituting the nodes, and the average monthly shared audience between the outlets capturing the weight of the edge between the corresponding nodes. I also construct the “augmented” network, by adding self-loops to every node of the “baseline” network where the weight of the self-loop is the average monthly audience for that node. Further, each node is manually annotated with the language in which the outlet primarily publishes. These annotations serve as the “ground truth” for appraising the performance of the algorithms, as we would expect outlets of the same type (i.e. ones that publish in the same language) to share heavier overlap with other outlets of the same type, than with outlets of different types. We would therefore expect the algorithms to assign outlets of the same type (i.e. corresponding to the same language) to the same community. In all, the network had 174 nodes and 14, 932 edges.

**Figure 5:**
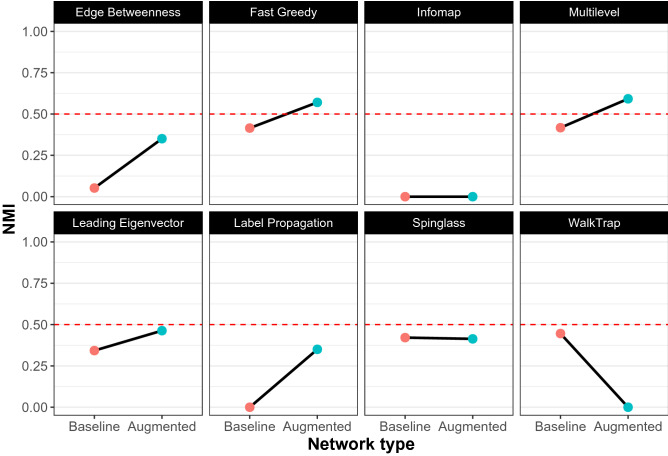
The performances of the 8 community detection algorithms are shown on the “baseline” (pink) and “augmented” (cyan) empirical network. The y-axis tracks the Normalized Mutual Information (NMI) value for each algorithm. As with the synthetic networks, Fast Greedy, Multilevel, and Spin-Glass outperform the others on the “baseline” network. The performances of FG and ML are yet again further enhanced with the augmented network, while that of WT is lowered. Overall, it is FG and ML on the augmented network, that perform the best.

**Figure 6:**
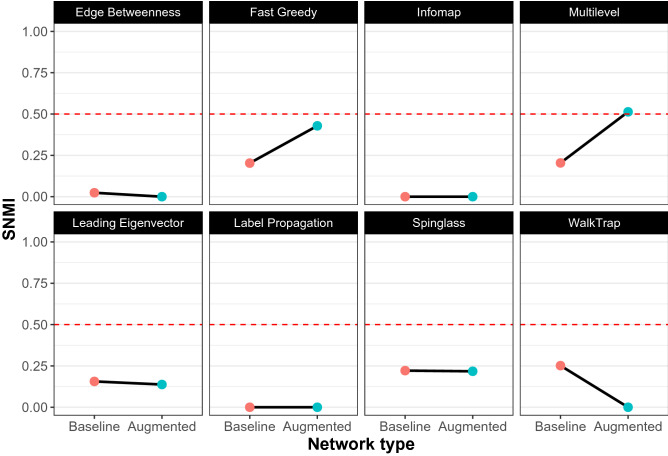
The performances of the 8 community detection algorithms are shown on the “baseline” (pink) and “augmented” (cyan) empirical network. The y-axis tracks the scaled Normalized Mutual Information (SNMI) value for each algorithm. As with the synthetic networks, Fast Greedy, Multilevel, and Spin-Glass outperform the others on the “baseline” network. The performances of FG and ML are yet again, further enhanced with the augmented network, while as before that of WT is lowered. Overall, and as before, it is FG and ML on the augmented network, that perform the best.

Figure 5 shows the results of running the eight algorithms on the “baseline” network and the “augmented” network in pink and cyan respectively. The y-axis tracks the Normalized Mutual Information score. When compared to the synthetic networks under conditions of moderate to high randomness, all the algorithms perfectly replicated their performances on the real world network as well. FG and ML out-performed the rest yet again, both working even better when the real world network was “augmented” with self-loops—achieving the two highest NMI scores. With scaled *NMI* values, the findings were qualitatively similar (see Fig. 6). In other words, FG and ML were better able to reveal the underlying linguistic fragmentation in the news consumption patterns of online Indian news consumers, better than any of the other algorithms.

## Discussion

Co-exposure networks and community detection algorithms are powerful analytical tools that help reveal insights into a variety of socio-political phenomena ^1–7^. Not only is the use of such techniques to model consumption intuitive, they allow for the precise operationalization of various social-scientific phenomena that had hitherto defied formal measurement. They help add richness to the quantitative description of media exposure patterns, and allow various macro-level and often emergent phenomena to be effectively modeled. While their wide usage in recent years in diverse contexts has helped cement their place in social science methodology, the manner of their application has often lacked formal justification. This has prevented prior findings from being contextualized within a robust methodological framework, as well as prevented findings from different studies in different contexts, which make different analytical choices, from being compared and consolidated systematically.

In this paper, I proposed a formal model for constructing co-exposure networks with a view to identify the most suitable community detection algorithm that could best reveal the underlying community structure reflecting selective exposure patterns. This model worked by simulating media exposure of a set of pre-programmed agents in an artificial media environment. This allowed me to construct large numbers of synthetic co-exposure networks, for various combinations of the model parameters. Eight community detection algorithms were then applied to these synthetically generated networks, and their performances were appraised with the use of the Normalized Mutual Information metric. I also proposed a methodological modification, informed by prior work ^25^, to further enhance the performance of these algorithms. This modification - which entailed adding weighted self-loops to every node in the network - enabled two of the eight algorithms, Fast Greedy and Multilevel, to significantly outperform the others in being able to better unveil the underlying selective exposure patterns that the model predicted. However, because a real world network may behave very differently from how artificial networks behave in theory, I repeated the comparison of the algorithms on a novel, large web-browsing network data set of a nationally representative sample of Indian internet users. Comparison of the algorithms on this empirical data showed that their performances on the artificial networks were faithfully replicated, with Fast Greedy and Multilvel significantly outperforming the others and yielding a high *NMI* score.

There are two key takeaways of this research. The first applies to future studies seeking to characterize selective exposure patterns by identifying the underlying community structure of co-exposure networks. My results suggest that Fast Greedy and Multilevel are the two most suitable algorithms for this purpose, that work remarkably well even in the presence of substantial noise, when many of the others fail to identify any community structure at all under similar conditions. For future researchers interested in using community detection in co-exposure networks to appraise selective exposure patterns, Fast Greedy and Multilevel could therefore hold great promise. As this study showed, augmenting co-exposure networks can produce significant improvements in their performances as well.

The second takeaway is more general, and contributes to the core novelty of this study. It demonstrates how a formal model, based on our theoretical understanding of a socio-political phenomenon, can be used to inform one’s analytical choices for capturing that phenomenon itself. In other words, a formal model can not just contribute to theoretical, but to methodological progress as well. By building a theoretical model of audience behavior and comparing the effectiveness of different algorithms in capturing audience dynamics, the study was able to make theoretical predictions regarding which algorithms work better than others in a specific category of complex networks. Validation of these theoretical predictions using empirical data demonstrated the utility of the exercise in informing analytical choices for studying the same phenomenon in the future. Of course, a model, by definition is a simplification of reality and there are many ways in which any model can be made better. While this set of simulations yielded promising results, I recognize that there are various parameters that could be further added to make this model even more complete and realistic. For instance, instead of a population level randomizing parameter $\rho$, one could use an agent-level randomizing parameter. The distribution of this parameter, could in turn be informed by empirical data of how selectivity in news consumption patterns is distributed in an audience population. Next, instead of having the same number of outlets and agents per type, as this model does, a future model could introduce more complexity by adding variability in these distributions, and then assess whether the findings qualitatively change. Finally, my model was limited by the availability of computing power in the number of outlets and agents I could simulate. Future studies can explore if these findings replicate for larger numbers of outlets, and more importantly, for larger numbers of agents. There is also the need to validate the findings from this model on other real world co-exposure networks. While the ComScore Indian dataset offered a useful empirical test, it remains to be seen if the community detection algorithms tested here behave similarly in co-exposure networks derived from other real world contexts such as social media. I invite other scholars to use this study to inform their own modeling exercises, and build on it in the future.

## Supplementary Information

Supplementary Information.
