# Supplementary material for: A systematic comparison of community detection algorithms for measuring selective exposure in co-exposure networks
Source: Sci Rep. 2021 Jul 26;11:15218. doi: 10.1038/s41598-021-94724-1 (PMC8313591; doi:10.1038/s41598-021-94724-1)
Supplement: Supplementary file 1 — Supplementary Information. [file 41598_2021_94724_MOESM1_ESM.pdf]

Supplementary Information for
A Systematic Comparison of Community Detection Algorithms for Measuring Selective Exposure in Co-exposure Networks

Subhayan Mukerjee[1]

[1]Department of Communications and New Media, Faculty of Arts & Social Sciences, National University of Singapore, Blk AS6, 11 Computing Drive, Singapore, 117416

Contents

I. Pseudocode **2**
- A. Pseudocode for Simulation . . . 2
- B. Pseudocode for Network Construction . . . 2

II. Sensitivity Analysis **3**
- A. Results for $\alpha = 3$ and $k = 1$ . . . 3
- B. Results for $\alpha = 3$ and $k = 15$ . . . 4
- C. Results for $\alpha = 1$ and $k = 3$ . . . 5
- D. Results for $\alpha = 2$ and $k = 3$ . . . 6

III. Robustness Analysis **7**
- A. Results for NMI using $variant$ = "min" . . . 7
- B. Results for NMI using $variant$ = "$sqrt$" . . . 9
- C. Results for NMI using $variant$ = "sum" . . . 11
- D. Results for NMI using $variant$ = "$joint$" . . . 13

IV. Direct comparison of NMI values for all algorithms **15**

V. Details about Empirical Network Construction **16**

I. Pseudocode

A. Pseudocode for Simulation

Algorithm 1 Simulation

procedure INITUNIVERSE(n_1, n_2, n_3)
    $M \leftarrow$ set of n_1 outlets $\{m_1, m_2, ..., m_{n_1}\}$
    $A \leftarrow$ set of n_2 agents $\{a_1, a_2, ..., a_{n_2}\}$
    $T \leftarrow$ set of n_3 types $\{t_1, t_2, ..., t_{n_3}\}$
    $R \leftarrow$ set of randomizing parameters $\{0, 0.1, ..., 1.0\}$

    for each outlet $m_i \in M$ *loop* :
        Assign m_i randomly to a type $t_i \in T$ such that at least one outlet is assigned to every type
        Assign reputation r_i to m_i where $r_i \in$ power law distribution $P(\alpha)$ with exponent α
    Normalize elements of vector $R = \{r_1, r_2, r_3, ..., r_{n_1}\}$ to $[0, 1]$

    for each agent $a_i \in A$ *loop* :
        Assign a_i randomly to a type $t_i \in T$ such that at least one agent is assigned to every type
        Assign a_i to visit v_i outlets where $v_i \in$ skewed normal distribution $N(\mu, \sigma, k)$
    Scale elements of vector $V = \{v_1, v_2, v_3, ..., v_{n_2}\}$ such that $max(v_i) = n_1$ and $min(v_i) = 1$

procedure SIMULATEAGENTBEHAVIOR(r)
    for each agent $a_i \in A$ *loop* :
        Random outlets $o_r \leftarrow$ weighted sample $r * v_i$ outlets from M with weights $\in R$
        Selective outlets $o_s \leftarrow$ weighted sample $(1 - r) * v_i$ outlets of type t_{a_i} with weights $\in R$
        Outlets visited by $a_i = o_i \leftarrow o_r \cup o_s$
    $O \leftarrow \{o_1, o_2, ..., o_{n_2}\}$

B. Pseudocode for Network Construction

Algorithm 2 Network Construction

procedure CONSTRUCTNETWORK(M, A, O, r)
    Define bipartite $G(M, A, E)$ over vertex sets $M \cup A$ such that $e \in E$ between a_i and m_j iff $m_j \in o_i$
    $G'(M, E')_{baseline} \leftarrow$ projection of $G(M, A, E)$ over vertex set M
    Define $G'(M, E')_{augmented} \leftarrow$ as $G'(M, E')_{baseline}$ with self-loop on m_i = number of agents that visited m_i

II. Sensitivity Analysis

In this section, I report the results for different values of the parameters α and k.

A. Results for $\alpha = 3$ and $k = 1$

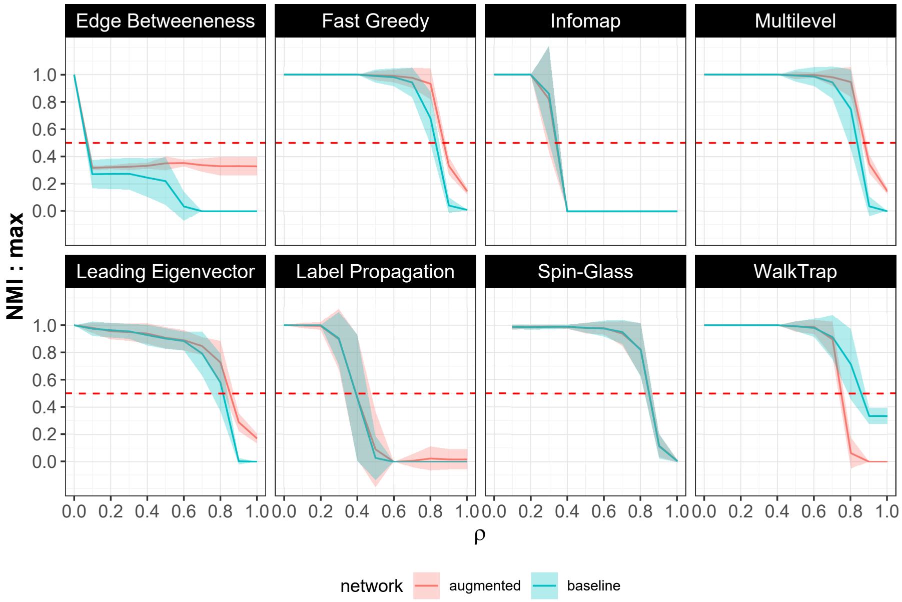

Figure 1: The performances of the 8 community detection algorithms are shown on the "baseline" (pink) and "augmented" (cyan) networks respectively. The y-axis tracks the Normalized Mutual Information value for each algorithm for every value of ρ, that determines the extent of randomness in the agents' behavior. The solid line tracks the mean NMI score for the 100 simulations for every value of ρ, while the ribbons indicate the standard deviation around the mean.

B. Results for $\alpha = 3$ and $k = 15$

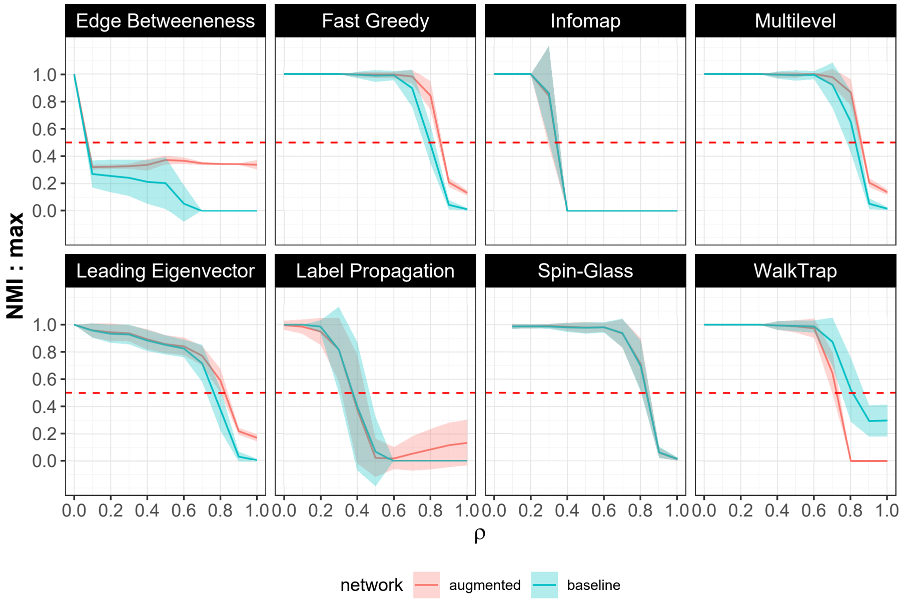

Figure 2: The performances of the 8 community detection algorithms are shown on the "baseline" (pink) and "augmented" (cyan) networks respectively. The y-axis tracks the Normalized Mutual Information value for each algorithm for every value of ρ, that determines the extent of randomness in the agents' behavior. The solid line tracks the mean NMI score for the 100 simulations for every value of ρ, while the ribbons indicate the standard deviation around the mean.

C. Results for $\alpha = 1$ and $k = 3$

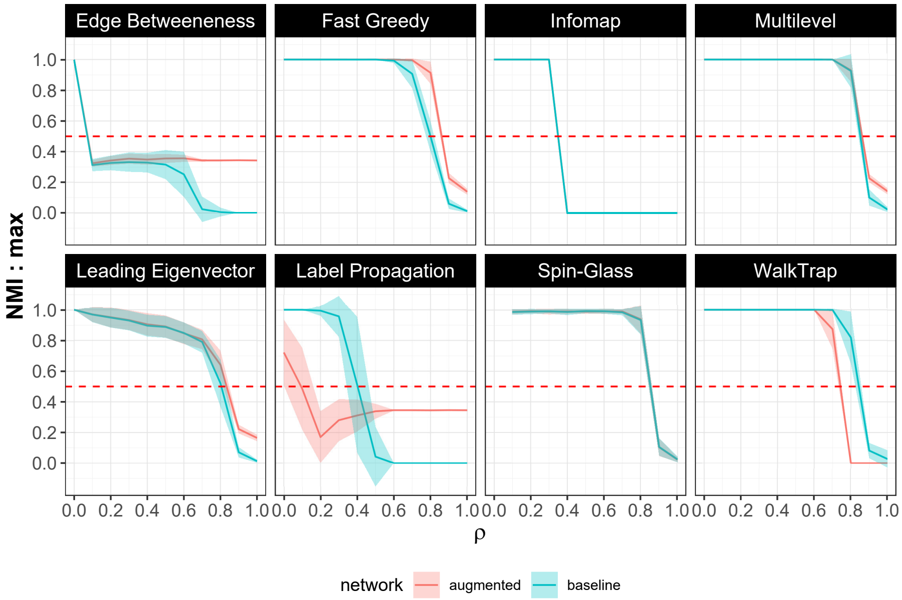

Figure 3: The performances of the 8 community detection algorithms are shown on the "baseline" (pink) and "augmented" (cyan) networks respectively. The y-axis tracks the Normalized Mutual Information value for each algorithm for every value of ρ, that determines the extent of randomness in the agents' behavior. The solid line tracks the mean NMI score for the 100 simulations for every value of ρ, while the ribbons indicate the standard deviation around the mean.

D. Results for $\alpha = 2$ and $k = 3$

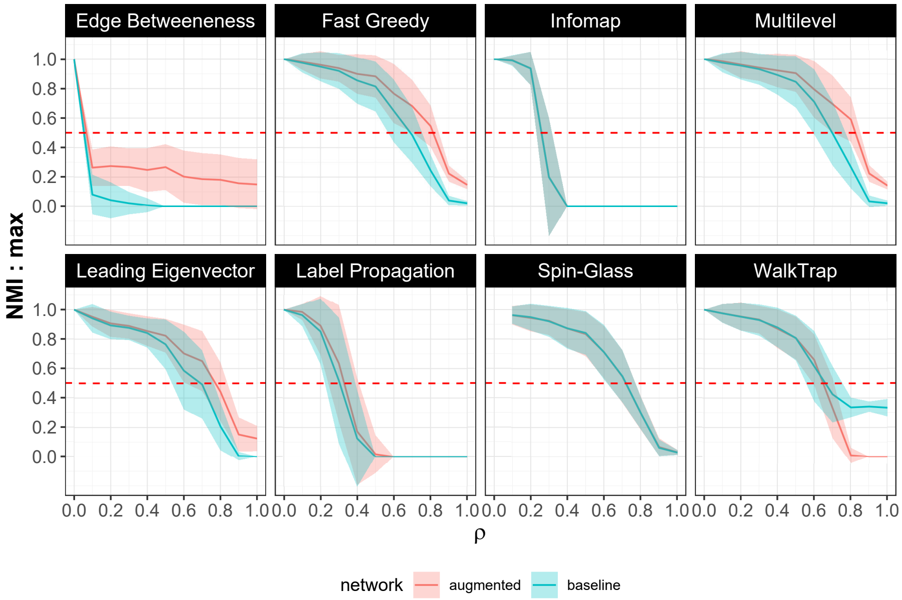

Figure 4: The performances of the 8 community detection algorithms are shown on the "baseline" (pink) and "augmented" (cyan) networks respectively. The y-axis tracks the Normalized Mutual Information value for each algorithm for every value of ρ, that determines the extent of randomness in the agents' behavior. The solid line tracks the mean NMI score for the 100 simulations for every value of ρ, while the ribbons indicate the standard deviation around the mean.

III. Robustness Analysis

In this section, I report the results for different variations of the NMI metric. Often, at high ρ values the NMI scores are inflated owing to the algorithm "incorrectly" finding large numbers of communities, by assigning each node to its own community. This is fixed by scaling the scores using a scaling factor that penalizes algorithms that identify large numbers of communities. The results using the scaled scores are also reported.

A. Results for NMI using $variant = \text{“}min\text{”}$

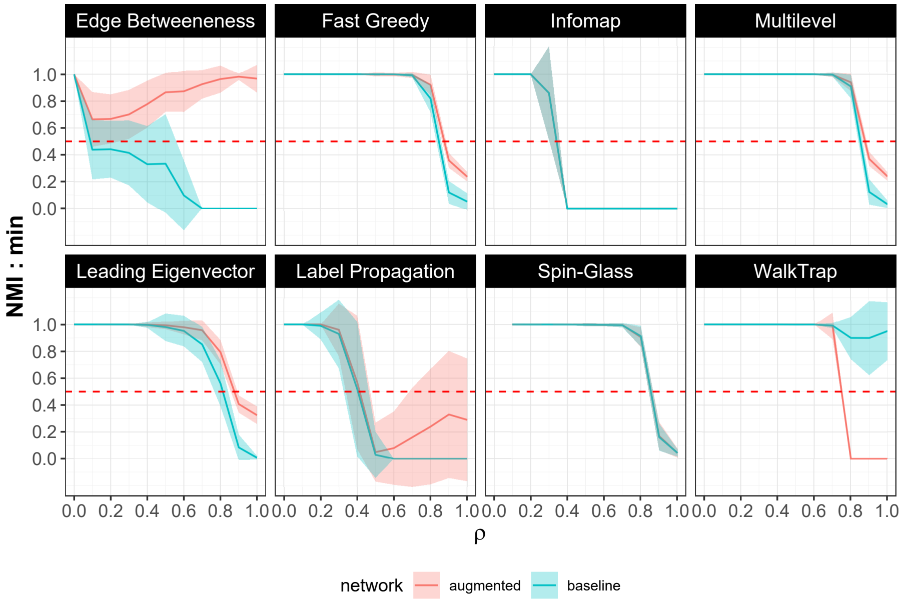

Figure 5: The performances of the 8 community detection algorithms are shown on the "baseline" (pink) and "augmented" (cyan) networks respectively. The y-axis tracks the Normalized Mutual Information value for each algorithm for every value of ρ, that determines the extent of randomness in the agents' behavior. The solid line tracks the mean NMI score for the 100 simulations for every value of ρ, while the ribbons indicate the standard deviation around the mean.

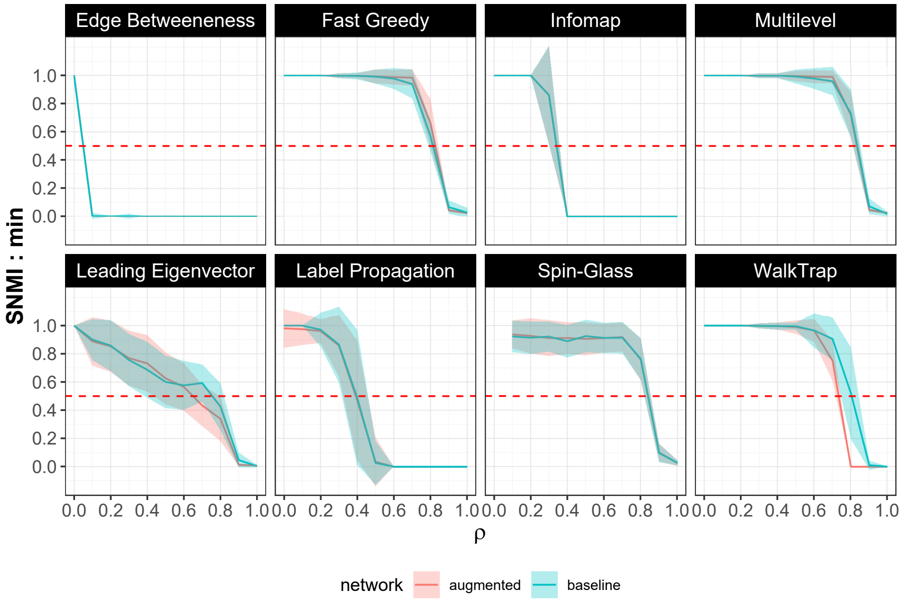

Figure 6: The performances of the 8 community detection algorithms are shown on the "baseline" (pink) and "augmented" (cyan) networks respectively. The y-axis tracks the scaled Normalized Mutual Information value for each algorithm for every value of ρ, that determines the extent of randomness in the agents' behavior. The solid line tracks the mean NMI score for the 100 simulations for every value of ρ, while the ribbons indicate the standard deviation around the mean.

B. Results for NMI using $variant = \text{"}sqrt\text{"}$

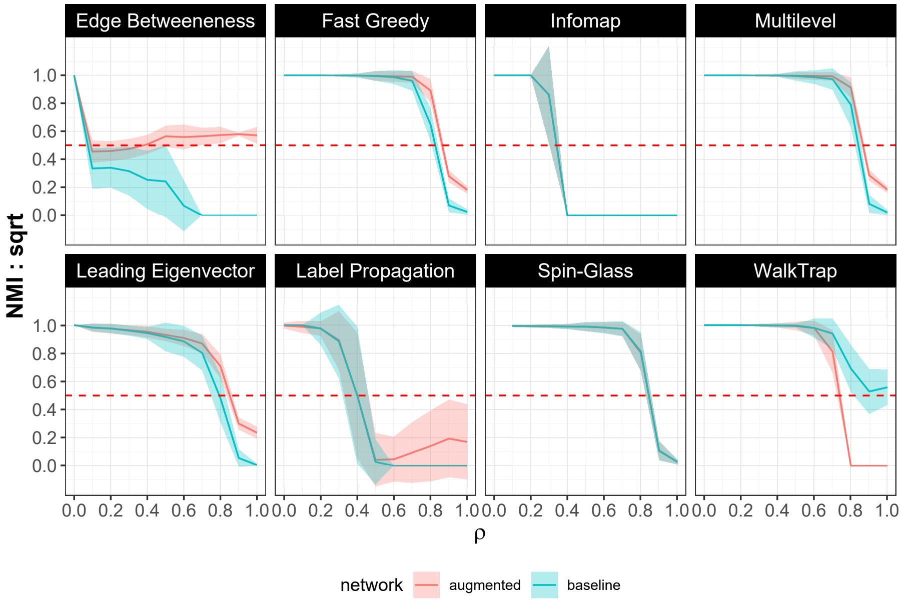

Figure 7: The performances of the 8 community detection algorithms are shown on the "baseline" (pink) and "augmented" (cyan) networks respectively. The y-axis tracks the Normalized Mutual Information value for each algorithm for every value of ρ, that determines the extent of randomness in the agents' behavior. The solid line tracks the mean NMI score for the 100 simulations for every value of ρ, while the ribbons indicate the standard deviation around the mean.

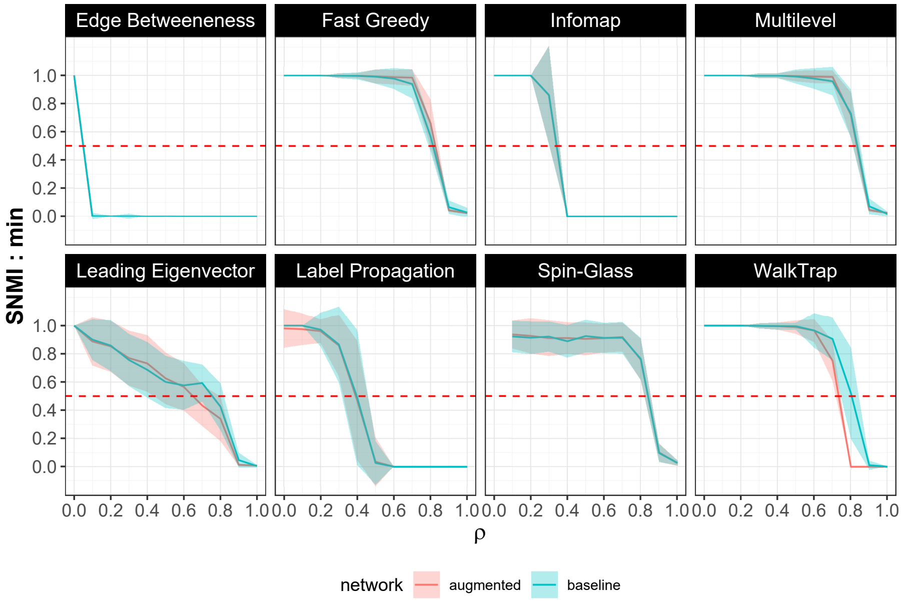

Figure 8: The performances of the 8 community detection algorithms are shown on the "baseline" (pink) and "augmented" (cyan) networks respectively. The y-axis tracks the scaled Normalized Mutual Information value for each algorithm for every value of ρ, that determines the extent of randomness in the agents' behavior. The solid line tracks the mean NMI score for the 100 simulations for every value of ρ, while the ribbons indicate the standard deviation around the mean.

C. Results for NMI using $variant = \text{“}sum\text{”}$

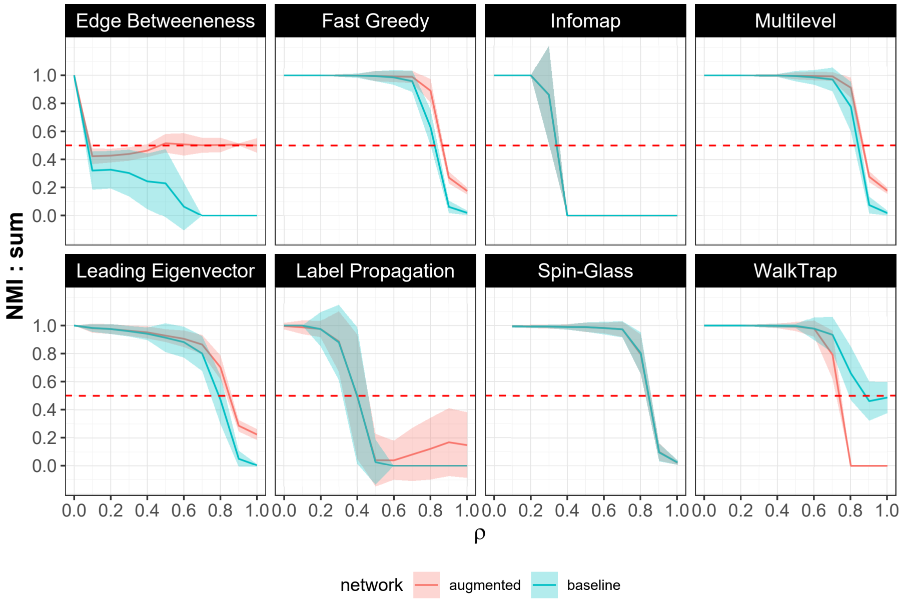

Figure 9: The performances of the 8 community detection algorithms are shown on the “baseline” (pink) and “augmented” (cyan) networks respectively. The y-axis tracks the Normalized Mutual Information value for each algorithm for every value of ρ, that determines the extent of randomness in the agents' behavior. The solid line tracks the mean NMI score for the 100 simulations for every value of ρ, while the ribbons indicate the standard deviation around the mean.

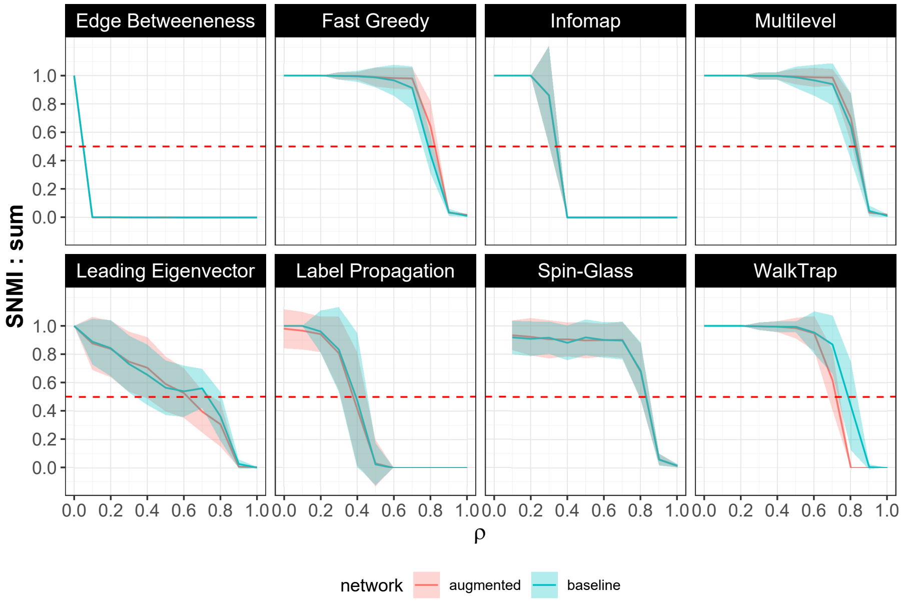

Figure 10: The performances of the 8 community detection algorithms are shown on the "baseline" (pink) and "augmented" (cyan) networks respectively. The y-axis tracks the scaled Normalized Mutual Information value for each algorithm for every value of ρ, that determines the extent of randomness in the agents' behavior. The solid line tracks the mean NMI score for the 100 simulations for every value of ρ, while the ribbons indicate the standard deviation around the mean.

D. Results for NMI using $variant = \text{"}joint\text{"}$

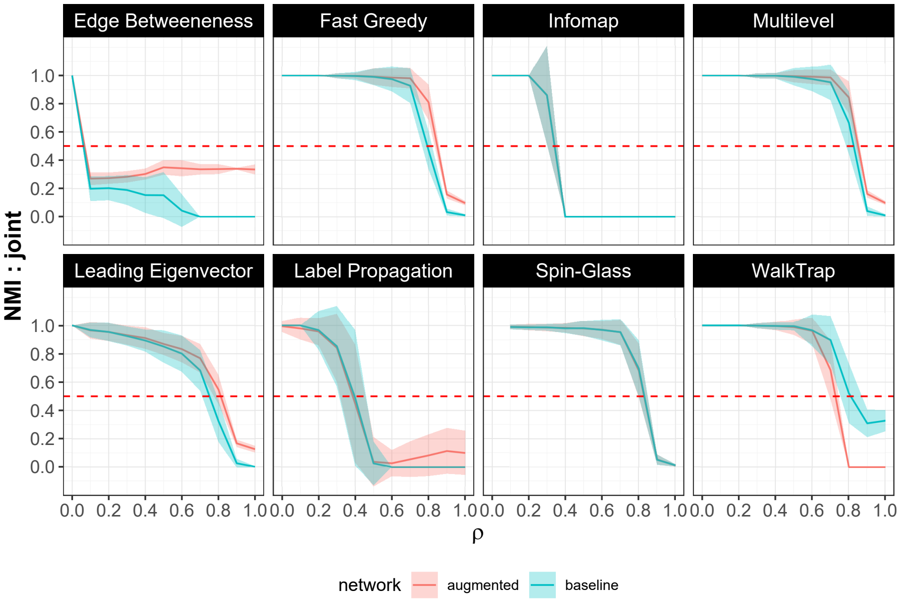

Figure 11: The performances of the 8 community detection algorithms are shown on the "baseline" (pink) and "augmented" (cyan) networks respectively. The y-axis tracks the Normalized Mutual Information value for each algorithm for every value of ρ, that determines the extent of randomness in the agents' behavior. The solid line tracks the mean NMI score for the 100 simulations for every value of ρ, while the ribbons indicate the standard deviation around the mean.

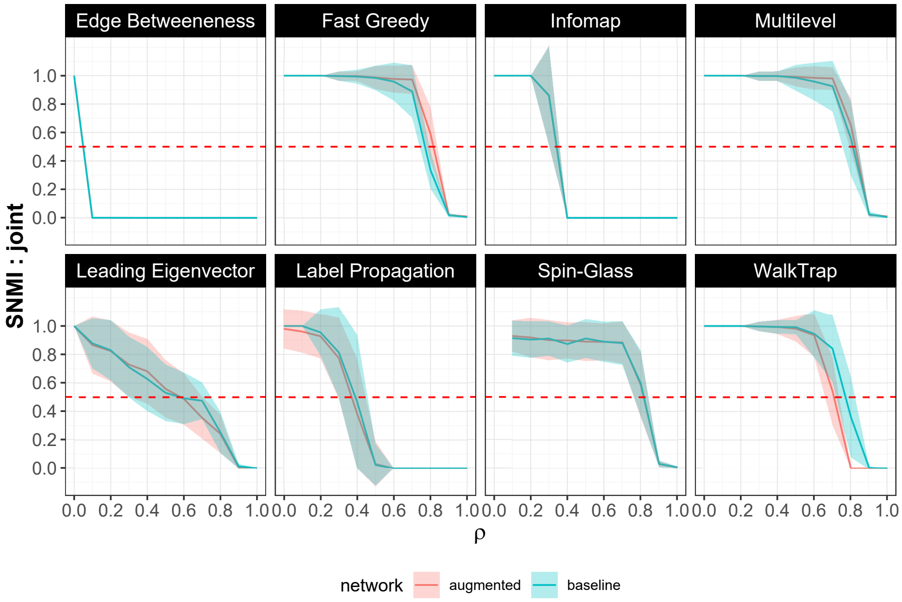

Figure 12: The performances of the 8 community detection algorithms are shown on the "baseline" (pink) and "augmented" (cyan) networks respectively. The y-axis tracks the scaled Normalized Mutual Information value for each algorithm for every value of ρ, that determines the extent of randomness in the agents' behavior. The solid line tracks the mean NMI score for the 100 simulations for every value of ρ, while the ribbons indicate the standard deviation around the mean.

IV. Direct comparison of NMI values for all algorithms

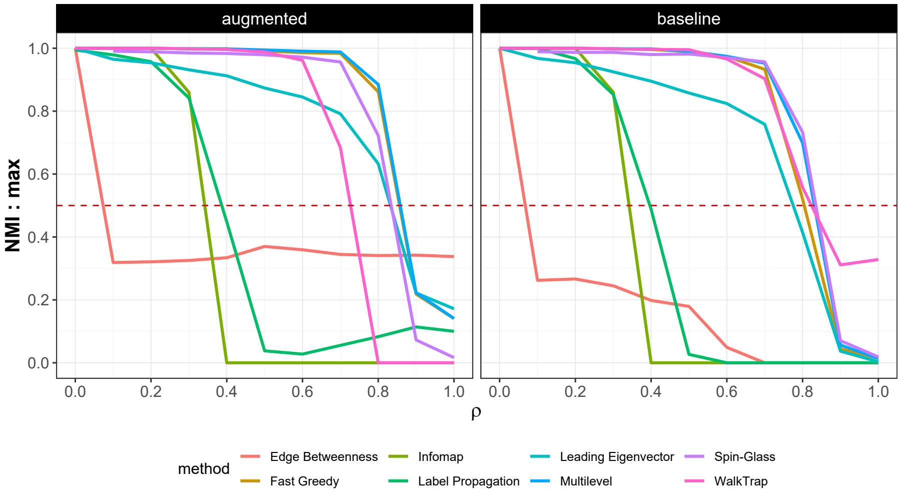

Figure 13: The performances of the 8 community detection algorithms are shown on the "augmented" (left) and "baseline" (right) networks respectively. All the algorithms are plotted on the same graph for direct visual comparison. The y-axis tracks the scaled Normalized Mutual Information value for each algorithm for every value of ρ, that determines the extent of randomness in the agents' behavior. The solid line tracks the mean NMI score for the 100 simulations for every value of ρ.

V. Details about Empirical Network Construction

For replication on an empirical dataset, a large scale dataset of desktop web-browsing behavior of Indian internet users (N > 50,000) was used. This dataset was obtained from the media analytics firm, ComScore. ComScore maintains representative panels of online users in over 40 different countries. They use a robust methodology called Unified Digital Measurement to integrate their panelists' data that they collect using a passive tracking software, with server side data that they capture by inserting specific tags in the source code of the web-pages. Their overall estimates, which are de-duplicated and then pre-processed to control for bot behavior, are then made available monthly. For the analysis, two statistics that ComScore provided were used: the first is the *total audience* of every news website (the unique number of visitors to a given website in a month), and the second is *cross visiting* for every *pair* of outlets (the unique number of visitors who visit a pair of websites in a month). These statistics were available over a 45 month period starting from October 2014 to June 2018. These data were used to first construct a "baseline" co-exposure network with media outlets constituting the nodes, and the average monthly shared audience between the outlets capturing the weight of the edge between the corresponding nodes. This "baseline" network was "augmented" by the addition of self-loops to every node where the weight of the self-loop is the average monthly audience for the node. Note that this is not a network of all possible media outlets in India, but of all the outlets that occurred every month for the 45-month period in the ComScore dataset. A website needs to register a minimum threshold of page views to be included in each month's data. Because the edge-weights of the network are monthly averages, including all the websites in the dataset would have resulted in biased estimates of the averages, as many websites did not occur in some of the months. The absence of these outlets in some months is because they did not have enough traffic to meet ComScore's minimum inclusion threshold. The resulting network was extremely dense with an edge density (without self-loops) of 0.9921
